# “Your Diet Defines Who You Are, Especially as a Man”: Masculinity in Online Media Focused on Healthy Eating for Men

**DOI:** 10.1177/15579883231213588

**Published:** 2023-12-21

**Authors:** Ruby Jelicich, Virginia Braun

**Affiliations:** 1 School of Psychology, Waipapa Taumata Rau/University of Auckland, Auckland, New Zealand

**Keywords:** healthism, neoliberalism, reflexive thematic analysis, hegemonic masculinity, men’s health crisis

## Abstract

In contexts marked by neoliberal ideology and a claimed “crisis” in men’s health, men are responsibilized to be/come healthy. Eating has long been a gendered practice in Western cultures, and recent cultural shifts have produced ways of eating that are both masculinized and (claimed) healthy. Online healthy eating advice, which encourages and supports men to eat healthily, is an important information source. However, such information draws on, reproduces, and/or disrupts existing meanings about men and eating. To understand contemporary representations of men and healthy eating, we examined 30 online media articles oriented specifically to this topic. Using reflexive thematic analysis from a social constructionist position, we developed two themes: *A lad’s looks and lifestyle* and *Mind over matter: The masculine mindset.* These themes together told an overarching story that healthy eating is effectively sold to men by drawing on traditional or hegemonic ideals of masculinity and effectively evoking access to an enhanced masculinity through healthy eating. While these representational practices may sell healthy eating to men, with likely positive health benefits, they also reinforce hegemonic ideals of masculinity which can be problematic from a health perspective.

## Men, Masculinity, and Healthy Eating

In this article, we build on and update previous research around media representations of men and healthy eating (e.g., Gough, 2006, 2007) in a shifted context of health considerations, constructions of *men’s* health (e.g., Crawshaw, 2007), and the rise of “wellness” diets some of which orient particularly to masculinity (such as paleo and keto). The framing of men’s health as in “crisis” has allowed for scrutiny and surveillance of men’s health practices, with pressure on men to change any “negative” health practices and adopt healthy ones (Gough, 2006; Mackenzie & Murray, 2021). Healthy eating is widely understood as a key health practice and something under individual control. But, given long-standing Western framings of “dieting” as a feminized domain (de Souza & Ciclitira, 2005; Mallyon et al., 2010), the task of orienting men to consider consumption is not *necessarily* a simple task. As media are a key source of health information (Dodds & Chamberlain, 2017; Pew Research Center, 2003; Wang et al., 2021), we sought to explore how healthy eating advice explicitly targeting men constructs healthy eating. Our analysis demonstrates that evocations to traditional ideas of masculinity are a key mode through which healthy eating is (still) being “sold” to men—a way to enhance masculinity.

Our ideas about health and the practices we engage in to foster good health are constructed within broader sociopolitical contexts (McDonald & Braun, 2022). Prior to the last three decades, “doing health” was considered a feminized endeavor in Western contexts, something men expressed little interest in, or concern about (Courtenay & Keeling, 2000; Lee & Frayn, 2008). Traditionally, idealized constructions of masculinity include characteristics such as heterosexuality, hypersexuality, strength, dominance, control, muscularity, rationality, and a lack of emotional display (Burton, 2014; Mallyon et al., 2010; Rosen & Nofziger, 2019). Theorizing around (“hegemonic”) masculinity as both idealized and strived for, but hard to reach and always under threat, positions (most) men as seeking to enhance their masculinity or masculine capital (Burton, 2014). Such gendered constructs have played a role in men’s apparent disinterest and reluctance to engage in health-related behavior (Clatterbaugh, 1990; Courtenay, 2009, 2000b; Nakagawa & Hart, 2019), and often operate counter to health (e.g., Mahalik et al., 2007). As discourses around what has become “men’s health” have shifted, men have opportunity and perhaps obligation to think of themselves in relation to health, in a way that does not automatically compromise their masculinity (Gough, 2006). One of the most prominent recent discursive shifts has been to construct men’s health as “in crisis” (Gough, 2006), something interpretable as not just ontologically true (Abubakar et al., 2015) but also rhetorical. The construction works rhetorically to shape society’s perception of men’s health as in need of urgent attention, both at the societal and individual levels. Media advice about health practices, such as what we analyze here, offers one means of “educating” men toward healthier lives.

Individual health needs to be understood in a wider context of neoliberal influence. Critical scholars around health have pointed to neoliberalism as a powerful shaping force for how individuals understand themselves and their relationship with health (e.g., LeBesco, 2011).^1^ Neoliberal ideology, which values and encourages individual choice, freedom, and autonomy, constructs people as responsible citizens who should consciously and consistently *manage* their health (e.g., Greenhalgh & Wessely, 2004; Lupton, 1999; Wardle et al., 2004), not least through engaging in “good” health practices (e.g., Crawshaw, 2007). Healthism has been described as an embodied and lived form of neoliberalism that constructs health as a high-priority goal that individuals should strive to achieve constantly; this state of “health” is also idealized, so it is not enough to simply *not* be sick (Crawford, 1980; McDonald & Braun, 2022; Scott, 2020). Healthist logic constructs such idealized health as an “achievable” goal, if the individual just makes the *right* lifestyle choices, actively engages with health information, and constantly works on one’s health (Crawford, 1980, 2006; Turrini, 2015). In influential media, men’s health has become framed in such terms (e.g., Crawshaw, 2007). The frameworks of neoliberalism and healthism position individual behavior as the most important factor influencing our health, obscuring the array of demographic characteristics and socio-structural constraints that may directly, or indirectly influence and inform an individual’s ability/inability to “be healthy” (Crawford, 1980; Hanganu-Bresch, 2020; McDonald & Braun, 2022; Parsons, 2016), and positions ill health as a consequence of “bad” personal or lifestyle choices (Overend et al., 2020).

In Western societies, eating (and food more generally) has become increasingly associated with health (Madden & Chamberlain, 2010), and positioned as a key mode through which health can be lost or gained (Burkert et al., 2014; Manoogian et al., 2019; World Health Organization [WHO], 2020). A social constructionist understanding of “healthy eating” positions it not as based in (simple) nutritional fact, but as an ontological object shaped by people, that shifts and changes over time and place; multiple “truths” of healthy eating can co-exist (McDonald & Braun, 2022). People's sense-making around healthy eating depends on social contexts, historical and current (Paquette, 2005; Ristovski-Slijepcevic et al., 2008), including constructions of gender.

Various studies have highlighted that healthiness has been less important to men than women, when considering food and eating (e.g., Courtenay et al., 2002; Fagerli & Wandel, 1999; Wardle et al., 2004; Wardle & Solomons, 1994). Individual men’s eating “choices” are made in a context not just of constructions of masculinity, but of gendered associations with foods themselves, where (some) foods are coded as masculine and feminine (see Beardsworth et al., 2002; McPhail et al., 2012; Roos et al., 2001)—for instance, a long-standing association of (red) meat with masculinity in Western cultures (Calvert, 2014; Carroll et al., 2019; Greenebaum & Dexter, 2018; Lyons, 2009). *Practices* associated with (healthy) eating are also gendered, such as home cooking positioned and practiced as a traditionally (hetero)feminine activity (Gough, 2007; Roos et al., 2001; Stibbe, 2004), and relational space (e.g., Mróz et al., 2011). With “dieting” similarly associated with femininity/women (Gough, 2007; Mallyon et al., 2010), a blurring between “dieting” and eating healthily situates healthy eating as a feminized activity (Mallyon et al., 2010). If idealized hegemonic masculinity is understood as oppositional to femininity, engaging in “feminized” healthy eating practices may be interpreted as a threat to masculinity (Greenebaum & Dexter, 2018). These studies suggest men who eat healthily (or want to) navigate a context where their masculinity is potentially at stake.^2^ Studies on other eating practices associated with femininity, such as veganism and weight-loss dieting (e.g., Mallyon et al., 2010; Mycek, 2018), have highlighted that men attempt to construct their eating practices around recognizably masculine modes, such as through rationalizing choices via scientific/expert research. Traditionally, masculine values of rationality and logic, rather than whim or emotion, are positioned as the basis for practices, rendering a particular way of eating *as* aligned with masculinity.

Media shapes what people know and understand (Favaro et al., 2017), for instance, in relation to health. Looking beyond simple causal influence models, the relationship between media representation, meaning-making, and health practices is complex and nuanced. Social media has allowed for an interactive and communicative space that disrupts traditional health information hierarchies, providing a space for constructions of health and eating that deviate from these traditional sources (Carruthers & Braun, 2020). Not only do media messages not necessarily align with “official” health advice, but they can also problematically reiterate wider societal norms, including gendered ones (Seale, 2003). Gough’s (2007) analysis of gendered constructions and representations of food, men, and health in UK newspaper articles, for instance, highlighted that these media evoked stereotypically gendered ideas to discuss men and diet and drew on hegemonic images of masculinity.

As lives are increasingly mediated (Favaro et al., 2017), media, which contain both explicit^3^ and implicit messages/information about topics like health and healthy eating (Seale, 2003), become a crucial site for analytic interest. The public considers media a primary provider of health and diet-related information (Dodds & Chamberlain, 2017), with online media one of the most popular sources for obtaining health information (Wang et al., 2021); searching for health or medical information online has been one of the most popular uses of the internet (Pew Research Center, 2003). Men recently ranked online media sources as one of their top two preferences for sourcing health information (Hodyl et al., 2020).

Analyses of media related to men and (healthy) eating have, until our study, focused exclusively on print media sources, such as newspaper articles and magazines. Stibbe’s (2004) critical analysis of a selection of *Men’s Health* magazines concluded that while the magazine articles contained aspects of helpful health information, their advice reproduced health ideals based on hegemonic masculinity, reinforcing male power, dominance, and, ironically, negative health behaviors—such as red meat consumption and convenience foods. Gough’s (2007) research with UK newspaper articles similarly identified representations around men and diet, which conformed significantly to hegemonic masculine ideals. The articles regularly depicted the “male diet” as unhealthy and restricted, across men from different social classes and reiterated the gendered construction of men as disinterested and unknowledgeable about (healthy) eating practices. Hegemonic masculine values of rationality, calculation, control, and logic (Gough, 2007; Mallyon et al., 2010) were evident both in the construction of food as fuel for more important activities, rather than a form of pleasure, and regular use of expert discourse, science, and statistics.

We aimed to extend the existing research on men and healthy eating in print media (e.g., Gough, 2007; Stibbe, 2004) by exploring contemporary healthy eating advice/information directed to men in online media. In a societal and media context that has changed significantly in the last 15 years, with the rise of social media and wellness cultures, as well as dietary modes directed toward masculinity, we sought to understand what meanings and messages are being made available to men around healthy eating, and about themselves *as men* in relation to healthy eating. Do the previously evidenced masculinizing discourses remain prominent, or are newer/different meanings evident around men and healthy eating?

## Method

We used a simple qualitative design, developing a robust media selection strategy and analyzing the resultant dataset using reflexive thematic analysis (TA; Braun & Clarke, 2022) from a social constructionist theoretical stance.

### Ethics

Online media articles (which constituted our dataset) have been understood as public spaces, both accessible to and designed for public use and engagement. When used as data, there are typically no ethical requirements for obtaining informed consent nor obligations to protect individual privacy (Convery & Cox, 2012; Favaro et al., 2017). In our case, this made the study exempt from institutional requirements for ethical review and approval.

### Dataset Generation and Evaluation

Our final dataset comprised 30 online, non-academic media sources, offering healthy eating information and advice for men. We wanted a dataset that would feasibly match the real-world experience of men searching for and reading advice and information around healthy eating. The dataset was generated and selected via a layered process. We first explored—then rejected—using search analytic tools to generate searches based on actual search terms—the combination of topic and location was too specific. We then decided on a strategy of multiple search phrases, using a private Google browser to prevent search history from influencing the results. As the *first* page of search results receives almost 95% of the web traffic (Kaye, 2013), we initially settled on only sampling the first two pages of hits per phrase. Our first two search phrases were “healthy eating for men” and “how to eat better as a man”; two subsequent ones were added based on Google’s “people also ask” suggestions from the “healthy eating for men” search: “what should a man eat to stay healthy” and “what food should a man eat.” We additionally sampled the third page of hits for our primary search term, “healthy eating for men.” Once duplicates had been removed, we had 58 media articles. These were evaluated for relevance to the research question, and excluded if they were not *specifically* targeted at men as eaters, not structured as healthy eating advice (e.g., just offered a sample meal plan), or were inadvertent double-ups. This review phase resulted in a final sample of 30 items (coded DI1-DI30; see Table 1).

**Table 1.** Media Dataset

Search term	URL	Data code
Healthy eating for men	https://www.everydayhealth.com/mens-health/building-a-healthy-diet.aspx	DI1
Healthy eating for men	https://www.eatright.org/health/wellness/healthy-aging/healthy-eating-for-men	DI2
Healthy eating for men	https://www.betterhealth.vic.gov.au/health/healthyliving/Mens-nutrition-needs	DI3
Healthy eating for men	https://www.onhealth.com/content/1/boost_male_health	DI4
Healthy eating for men	https://www.bbcgoodfood.com/howto/guide/balanced-diet-men	DI5
Healthy eating for men	https://www.healthyfood.com/advice/healthy-eating-for-men/	DI6
Healthy eating for men	https://www.fitfatherproject.com/healthy-eating-for-men/	DI7
Healthy eating for men	https://familydoctor.org/men-eat-right-stay-healthy/	DI8
Healthy eating for men	https://www.eatthis.com/best-foods-for-men/	DI9
Healthy eating for men	https://www.eatthis.com/healthy-eating-habits-for-men/	DI10
Healthy eating for men	https://youngmenshealthsite.org/guides/healthy-eating/	DI11
Healthy eating for men	https://www.webmd.com/men/ss/slideshow-foods-to-boost-male-health	DI12
Healthy eating for men	https://www.webmd.com/healthy-aging/best-diets-for-men-over-50	DI13
Healthy eating for men	https://www2.illinois.gov/cms/benefits/StateEmployee/BeWell/FoodForThought/Pages/Food_Healthy-Men_June2021.aspx	DI14
What should a man eat to stay healthy	https://www.today.com/health/7-healthy-nutritious-foods-men-should-eat-longer-life-t182927	DI15
What should a man eat to stay healthy	https://community.jennycraig.com/healthy-habits-blog/eat-well/healthy-diet-men	DI16
How to eat better as a man	https://www.mensjournal.com/food-drink/20-essential-superfoods-for-every-mans-diet/	DI17
What food should a man eat	https://www.eatthis.com/foods-men-eat-every-day/	DI18
What food should a man eat	https://www.mensjournal.com/food-drink/best-worst-foods-man-can-eat/	DI19
What food should a man eat	https://www.realbuzz.com/articles-interests/nutrition/article/10-foods-all-men-should-eat/	DI20
What food should a man eat	https://coach.nine.com.au/diet/11-foods-men-should-eat-every-day/807c9a14-a715-442a-8cdc-5b35ec695205	DI21
What food should a man eat	https://food.ndtv.com/health/21-best-foods-that-all-men-should-eat-1740938	DI22
What food should a man eat	https://www.menshealth.com/uk/nutrition/a32852405/foods-men-should-eat-more/	DI23
What food should a man eat	https://www.newshub.co.nz/home/lifestyle/2020/06/the-top-ten-foods-men-should-be-eating-more-of-for-good-health.html	DI24
Healthy eating for men p3	https://health.usnews.com/wellness/food/articles/best-diets-for-men	DI25
Healthy eating for men p3	http://www.knowitallnutrition.com/how-to-help-your-family-enjoy-nutritious-foods/	DI26
Healthy eating for men p3	https://timesofindia.indiatimes.com/life-style/food-news/healthy-eating-tips-for-men-of-all-age-groups/photostory/92385485.cms?picid=92385734	DI27
Healthy eating for men p3	https://www.menshealth.com/nutrition/a19546197/healthy-grocery-list-for-men/	DI28
Healthy eating for men p3	https://fitlifefanatics.com/healthy-foods-for-men/	DI29
Healthy eating for men p3	https://www.healthyfood.com/advice/how-to-man-tain-your-health/	DI30

Overall, the size and adequacy of the dataset were guided and assessed in relation to the notion of information power (Malterud et al., 2016). In contrast to the widely used—but not methodologically appropriate (see Braun & Clarke, 2021b)—concept of saturation, information power suggests dataset size should be based on a consideration of the information *richness* of the data in relation to the study’s aim, scope, and approach (Malterud et al., 2016). We took this question into the first analytic phase of familiarization (Braun & Clarke, 2022), which indicated sufficient depth and richness to explore our research question in-depth.

### Analytic Approach and Process

We used reflexive TA from a social constructionist theoretical standpoint to analyze the data (Braun & Clarke, 2022). Within a constructionist framework, the way we understand the world is culturally and historically situated, a product of how the world is (re)presented and (re)produced through language and discourse, rather than reflecting some external reality or truth (Burr, 2015). Taking this standpoint when using reflexive TA meant we explored the data, asking what socially available discourses, meanings, and ideas about healthy eating for men were (re)produced there, through the patternings we came to understand through our analytic process (see also McDonald & Braun, 2022), with interest in the implications of the constructions we explored.

Analysis followed the broad processes for reflexive TA (Braun & Clarke, 2022). In the first phase of analysis, familiarization, Author 1 (Ruby) read and re-read the data, making initial notes of observations, thoughts and questions, which were then discussed with Author 2 (Ginny). The most notable observations at this point were that the media articles appeared to construct healthy eating in ways that sell healthy eating to men, and they seemed to do so by linking and discussing healthy eating in relation to traditionally masculine ideals, including sexual virility, body aesthetics centered around muscles and scientific discourse. These insights were taken into, but did not limit, how we tackled the next phase, coding.

The coding was done in *Microsoft Word*, with each data item entered into a table with a column for codes. Our coding followed a robust layered process. Ruby, in consultation with Ginny, comprehensively coded each article twice, mixing up the dataset order for the second round of coding to disrupt any familiar flow that had developed in the first round of coding, and to ensure that all data items got a similar depth of engagement and insight (Braun & Clarke, 2022). The approach combined a mix of inductive and deductive orientations, with concepts like hegemonic masculinity (Connell & Messerschmidt, 2005) providing a partial lens through which we interpreted and made sense of some of the data. Codes shifted between semantic and latent—some, such as “healthy eating enhances men’s fertility,” captured surface-level meaning; others, such as “healthy eating is not natural” captured meaning at a deeper or more implicit level. Ruby did a third round of coding to check for new areas to code, which resulted in few additions, and a final list of approximately 450 codes.

For initial theme generation, we used *Miro*, an online whiteboard, to start to cluster codes visually. Clustering was informed by Ruby’s familiarization observations/understandings; while clustering, we were considering different constructions around healthy eating that brought together different codes and seemed to *sell* healthy eating to men. Given familiarization observations about masculinity, we had tropes of (hegemonic) ideals of masculinity as a guiding interest, but allowed space to develop other clusters of shared meaning that did not link clearly to Ruby’s familiarization observations. This process, led by Ruby and reviewed with Ginny, led to code clusters centered around how healthy eating was constructed as enhancing men’s looks, health, life performance, fitness abilities, youthfulness, and sexuality. Further clusters centered around how healthy eating required self-control, demonstrated rational thinking, and more. We went beyond our initial observations, and developed clusters centered around men’s eating/health habits and knowledge, as these were prominent throughout the data and resonated from existing research; ultimately on review, these latter became contextual information rather than themes, as part of the analytic story.

With further clustering and consideration (led by Ruby, and in review with Ginny) validating our initial impression that there was an important analytic story based around “selling” healthy eating through evocations to (hegemonic) masculinity, some code clusters shifted out of the analytic development, and others were revised. At the end of this phase, we had clusters that we could separate into two broad areas: clusters around self-discipline/control and rational/logical thinking, united because they seemed to be centered around internal characteristics; clusters around sexuality, youthfulness, body aesthetics, life performance, and fitness abilities, united around more external characteristics. With these two clear initial themes, we moved into the next phases of development, refinement, and naming, and then writing (Braun & Clarke, 2022).

This process remained led by Ruby, in consultation with Ginny. The review process of going back to the dataset confirmed the themes were relevant and captured an important and interesting analytic story. However, we reviewed and revised potential subthemes (initially developed around the code clusters), both to reduce overlap between subthemes, and prevent the themes themselves from being too fragmented (Braun & Clarke, 2021a). Our final themes were: *A lad’s looks and lifestyle*—which included the subthemes “*shrink-wrapped abs and three-dimensional arms”* (DI17); “*magic for your manhood” (DI9)*; and *the aging antidote*—and *Mind over matter: The masculine mindset*—which included the subthemes *manly motivation* and *the disciplined dude*.

Reflexive TA is situated within an understanding of research as an inescapably subjective process, requiring reflexivity in both research process and writeup (Braun & Clarke, 2013, 2022). This reflexivity was both individual and collaborative. It involved Ruby keeping a reflexive journal and considering how her background in gender studies shaped her engagement with the data, and the knowledge produced through this research. Both researchers’ interest in masculinities and gender more broadly informed the lens through which we engaged with the data. Analytic interpretation was collaboratively interrogated between Ruby and Ginny; in data-oriented engagement with other postgraduate students at different points, new and different insights into the data provided useful checks for the developing analysis, as well as validation of the strength of the masculinity story.

## Analysis

Before discussing the two key themes—*A lad’s looks and lifestyle* and *Mind over matter: The masculine mindset*—we highlight some key elements of the dataset, to contextualize and situate our analysis. Previously identified discourses that construct men as not typically or naturally inclined/engaged with eating healthily but needing to be (e.g., Gough, 2007) remained pervasive in the dataset, positioning *unhealthy* eating as the default option and desirable through being tempting, easy, fast, and a craving (see also McDonald & Braun, 2022). The media items consistently positioned healthy eating as *unnatural* to men by suggesting that healthy eating requires (new) habits to be developed, prioritized, remembered, and (consciously) maintained, and offering strategies to support this (implicitly, and sometimes explicitly, difficult) task. The data often claimed or evoked a wider discourse of a “crisis” in men’s health (Gough, 2006), for instance, by referencing men’s health disparities, or situating men as tending to engage in health-harming behaviors, or lacking concern for their health. In a context where neoliberal ideology is pervasive (McDonald & Braun, 2022), the positioning of men’s health in “crisis,” both in the data and more broadly in dominant discourses of men’s health (see Gough, 2006), situates engaging in health practices such as healthy eating as an important or even essential practice for men. The *essentialness* of healthy eating was also explicitly referenced: “good nutrition is critical for good health” (DI8). This discourse of a crisis in men’s health, alongside the argued importance of, yet reported limited engagement in, healthy eating by men, provides context for our understanding of the dataset as constructing healthy eating in ways that should appeal to, and therefore *sell*, healthy eating to men.

As we would expect, healthy eating was framed explicitly and consistently as a health-enhancing practice, something that “can boost your health . . . and provide more energy” (DI29), both generally and as disease prevention, with items often noting “specific foods that don’t just improve men’s health generally, but also help stave off cancer, reduce the risk of heart disease, and boost overall energy” (DI1). However, the more interesting and nuanced analytic story was the ways healthy eating was more implicitly “sold” to men as something they should (want to) engage in, by drawing on hegemonic ideals of masculinity. This story evoked an enhanced *masculinity* for men who ate healthily, rather than simple health benefits. This framing was prevalent—two data items even discussed masculinity benefits ahead of health or longevity: one highlighted “the best foods for your penis” before “foods for your heart” or “foods that can keep cancer at bay” (DI9); another (DI6) discussed in depth the benefits of healthy eating on men’s work, social, sexual and home life, before discussing longevity. A causal link between healthy eating and masculinity enhancement was evoked relatively *implicitly* throughout the data, although not always: “there are no doubts about it—your diet defines who you are, especially as a man” (DI29).

We now discuss two themes which captured two modes of and for masculinity enhancement offered by eating healthily—one focused around external aspects (*A lad’s looks and lifestyle*), the other around more internal characteristics (*Mind over matter: The masculine mindset*).

### A Lad’s Looks and Lifestyle

This theme focuses on the ways healthy eating was sold to men through being positioned as improving traditionally masculine physical or external characteristics or behaviors. Across this broad pattern, we identified three notable repeated areas of improvement—sexual virility, a toned and muscular physique, and youthfulness. We report these in the subthemes “*Magic for your manhood”* (DI9), “*Shrink wrapped abs and three-dimensional arms” (DI17)* and *The aging antidote*.

#### “Magic for Your Manhood”

Sexual virility has been a core component of (hegemonic) notions of masculinity, connected to power and prestige (Burton, 2014). The data evoked this association by constructing healthy eating as a practice which can enhance men’s sexual virility—here virility encompasses men’s sexual *and* reproductive abilities. Healthy eating was positioned as a practice that can both prevent or reduce (risk of) fertility issues and sexual dysfunction, and as something that could enhance implicitly normal fertility and sexual performance. For example, the idea that fertility could be enhanced was often evoked when talking about specific foods or micronutrients:

By consuming zinc through cooked seafood, men can boost their fertility . . . (DI20)

Pine nuts are also a good source of magnesium, which boosts testosterone and helps keep sperm healthy and viable. (DI9)

Fertility was positioned as under threat if men did *not* practice healthy eating. For instance,

You only need a tiny bit [of selenium] for healthy sperm, but a tiny deficiency can be catastrophic for reproductive health. (DI9)

Zinc levels below normal are linked to poor sperm quality and male infertility. (DI12)

In a context where the average man was typically positioned as not eating healthily by nature, the risk to fertility through inattention to healthy eating—and micronutrients—becomes a clear and present danger for men. Engaging in healthy eating practices becomes the man’s tool for preventing harm to fertility. With fertility understood as a hegemonically masculine characteristic (Burton, 2014), through evoking fertility, healthy eating is presented to men as a way to enhance their masculinity, and unhealthy eating becomes a threat to masculinity.

A link between hegemonic masculinity and fertility has typically been more connected to men’s ability to impregnate women than to fatherhood (Mason, 1993)—and indeed, our data situated fertility *per se* as the (masculinity) benefit of healthy eating, only rarely referencing fatherhood:

In addition to fighting off colds, this chemical is necessary for male fertility. So, if you want to become a father, you may want to pack a handful of brazil nuts in your lunch. (DI4)

Want to become a dad? Pick up a few bags of baby carrots. (DI9)

Fertility orients to a future *outcome* as evidence of masculinity; a more immediate measure was through sexual *performance*—most notably in the data as evidenced by erectile capacity. For example,

What’s more, eating enough pistachios could give you more reliable and longer-lasting erections. (DI4)

[blueberries] also come with high levels of vitamins C and K. Besides, they help the erectile function as well and taste great. (DI29)

Here, the data evoked long-standing connections between (hegemonic) masculinity and sexual prowess (Mason, 1993; Oliffe, 2005; Thompson & Barnes, 2013). Given dominant understandings of men’s sexuality, and (hetero)sex more generally, which place the (erect) penis at the center of sex, enhancing erectile function operates as both sexuality and masculinity enhancer (e.g., see Potts, 2000). Representations around healthy eating for men that claim erectile benefits, draw on these sociocultural associations to sell healthy eating as masculinity enhancement. A discussion of benefits related to sexual virility has also been identified in popular media texts related to veganism—which lacks the masculinity-component of (red) meat (Johnson, 2011).

Similarly to fertility, discussion of erectile benefit from healthy eating *also* oriented to preventing future “erectile dysfunction”—or minimizing its impact. For example,

Blueberries have lots of vitamin K, which helps your blood clot, and plenty of vitamin C like most berries. But they also may help prevent or improve erectile dysfunction. (DI4)

A study published in the International Journal of Impotence Research discovered that pomegranate juice, which is rich in antioxidants that support blood flow, can help improve erectile dysfunction. (DI9)

We have highlighted the way masculinity through sexual virility was evident across the data, both as enhancement, and to ward off a future threat (or reduce current impact). The data both drew on and contributed to the wider discourse and construction of masculinity, where sexual performance and prowess are core for “better” masculinity (Oliffe, 2005). Sexual performance becomes one part of the wider constellation of evocations of masculinity that sold healthy eating to men as desirable practice, in our dataset.

#### Shrink-Wrapped Abs and Three-Dimensional Arms

Healthy eating was positioned as a means for preventing “unintended” (DI25) or “unnecessary” (DI16) weight gain—“maintaining a healthy diet can prevent weight gain or health issues” (DI13)—as well as weight and loss, and general weight management:

. . . eating well not only gives your body the nutrients it needs. It also helps keep weight under control. (DI8)

Spinach will prevent cancer, help in weight loss, reduce blood sugar and keep the body relaxed. (DI29)

Like a marathoner stretching before the big run, eating half a grapefruit before a meal can enhance your body’s fat-burning performance. A study published in the journal Metabolism found that this "warm-up" tactic can help whittle your middle—by up to an inch—in just six weeks. (DI9)

In the context of the dataset, weight, weight loss and weight management were connected both to health, and to masculinity. Traditionally coded as feminine concerns (Mallyon et al., 2010), these were (re)positioned as masculinity *enhancers*, enabling men’s conformity to hegemonically masculine body ideals (Gough, 2007; Mallyon et al., 2010), including “staying lean” (DI4) or obtaining a “defined” (DI9) physique for the man who does not already have the ultimate masculine embodiment of a toned, defined body (Martin & Govender, 2011; Ricciardelli et al., 2010). While discussion on weight loss and weight management alone may not suggest masculinity as key in selling healthy eating to men, the combination of this with claims of healthy eating as enhancing men’s ability to build and maintain muscle, situated healthy eating as a mode for masculinity. Healthy eating was positioned as a way for men to enhance their physique, particularly to facilitate muscle building, muscle maintenance, and fat loss. For instance, the building of muscle was regularly noted as a by-product or consequence of healthy eating:

Protein is essential. It helps you build and maintain hard muscle. (DI23)

Although technically a seed, millet should be treated as a grain. It helps enhance blood flow to your muscles thanks to its rich magnesium content, allowing them to develop and take on a more jacked look. (DI9)

With societal shifts in which men’s bodies have become objects of public scrutiny, not least through media representation (Gill, 2008), iterations of idealized or hegemonic masculinity since the mid-1980s (Edwards, 2016; Mort, 1988; Nixon, 1996; H. Pope et al., 2000; H. G. Pope et al., 1999) have emphasized appearance (Lanzieri & Hildebrandt, 2011), with muscular and toned physique, interpreted as the physical embodiment of characteristics such as dominance, power and strength (Parasecoli, 2005). Consistent with this, the data presented healthy eating as supporting the development of physical characteristics, such as “shrink-wrapped abs and three-dimensional arms” (DI17). Engaging in healthy eating—*for muscle*—was positioned as a way to enhance men’s masculinity, by increasing conformity to hegemonically masculine body ideals.

#### The Aging Antidote

With a potential inverse relationship between hegemonic masculinity and aging (Spector-Mersel, 2006), we theorized repeated claims around an extension of youth, as “how a man eats throughout his life can predict how well (or not) he ages” (DI26), as a third way healthy eating was constructed as enhancing masculinity. Reference to healthy eating enhancing or preventing a decline in men’s physical and mental functions/capabilities evoked eating as a means to slow or delay an implicit (sometimes explicit) *decline* that was associated with aging:

. . . in a study among older adults, the participants who ate one serving of spinach (or any leafy green) per day showed less cognitive decline than those who shunned greens- their cognitive abilities were similar to those of folks 11 years younger than them. (DI15)

Your diet can help fight disease and keep you looking and acting younger. (DI26)

By evoking anti-aging discourse, the data positioned aging and deviations from (the appearance of) youth as undesirable, and ideally resisted, and resistible, through healthy eating. One data item—evoking another masculinity trope—noted that without eating healthily, aging can lead to your body “rusting like a junkyard car” (DI19). In two items, healthy eating was positioned explicitly as a way to help one’s body “fight” (DI28; DI29) the aging process. Here, aging becomes not inevitability, but an unwanted invasive force, to be resisted through the right kind of eating. Many of the features associated with hegemonic masculinity, and promoted within the data through healthy eating, such as sexual virility and a toned and muscular physique, are associated with youth and often negatively correlated with age, a position articulated explicitly in some data items: “once you pass the age of 50, it’s natural for muscle mass to decrease over time” (DI13); “[as we age we involuntarily lose] muscle mass and muscular function” (DI27). Through claims of healthy eating extending youth, the dataset evoked an enhancement of masculinity by staying physically and mentally “younger” for longer.

### Mind Over Matter: The Masculine Mindset

Our first theme, *A lad’s looks and lifestyle*, centered on the ways the data evoked an enhancement of masculinity through healthy eating with an orientation to physicality—sexual virility, physiques, and youthfulness. Our second theme, *Mind over matter: The masculine mindset*, captures another key pattern in the way masculinity was positioned as facilitated by healthy eating. Here we focus on traditionally masculine internal or psychological attributes, considering the ways ideas of rational thinking and self-control/discipline were deployed to promote healthy eating to men, and were (thus) positioned as *demonstrable through* healthy eating. We highlight these through two distinctive subthemes: *Manly motivation* and *The disciplined dude*.

#### Manly Motivation

The data constructed healthy eating as a *rational* choice through the consistent and pervasive deployment of empirical/scientific evidence, scientific jargon, and expert opinion to bolster the data’s claims about the benefits and importance of healthy eating. For example,

Research shows that people who eat more cruciferous vegetables have a lower risk of prostate, lung, and colon cancer, says the National Cancer Institute. (DI28)

Multiple studies show that reaping a bigger portion of our protein from plants cuts instances of heart disease. (DI23)

One study showed that by substituting white rice for brown, you can lower your risk of developing type 2 diabetes. (DI4)

Through the deployment of scientific discourse, healthy eating is positioned as supported by, and based on, science (see also Mackenzie & Murray, 2021), deemed a superior form of healthy eating information (Dodds & Chamberlain, 2017). The data also constructed healthy eating as a *rational* choice through scientific jargon and displayed expertise. By scientific jargon, we refer to the use of complex scientific language, which deviated significantly from the tone and language of the rest of the data (which seemed more directed to its implied target audience of the “average man” [DI25]). For example,

On consumption, ellagitannins (ET), antioxidants abundant in pomegranate juice, break down to metabolites known as urolithins. Urolithins appear to inhibit the growth of human prostate cancer cells. (DI20)

Some data items also deployed expertise through quoting professionals (see also Dodds & Chamberlain, 2017), whose expertise was displayed with qualifications and/or professional titles, such as:

“Quinoa is also a great source of fiber and B vitamins,” says Christopher Mohr, Ph.D., R.D. a professor of nutrition at the University of Louisville. (DI17)

Through the use of science rhetoric and “expert” advice and opinions, healthy eating becomes the “choice” a right-thinking man would make—and within a neoliberalized healthist context, making right choices are part of doing health for men (e.g., Crawshaw, 2007). With science constructed as objective and conveying facts, a choice (to eat healthily) becomes situated within the domain of rational thinking/decision-making (Mycek, 2018). Scientific evidence has similarly been drawn on in men’s justifications for eating vegan or vegetarian (Banyte et al., 2022; Mycek, 2018) and for participating in weight-loss diets (Mallyon et al., 2010). Western traditions have long situated emotion-based decision-making as feminine, while rational, logical (“science” thinking) has been understood as masculine (Crawford et al., 1992; Petersen, 1998). By drawing on scientific discourse, and evoking traditionally masculine decision-making values, the data situated healthy eating *as* a masculine practice, (re)claiming what have typically been more feminized eating practices and considerations as legitimately the stuff of men. In a wider data context where men were normatively positioned as (still) *not* eating healthily, with such associations, engaging in healthy eating can itself evidence a psychologically masculine characteristic and decision-making process, displaying (to self or others) a kind of enhanced masculinity.

#### The Disciplined Dude

Healthy eating was depicted as a practice that requires self-control and discipline, a practice of restraint, restriction and moderation compared to implied normal eating (see also McDonald & Braun, 2022). Data items consistently deployed words such as “cut back” (DI1), “limit” (DI3), and “skip” (DI10) when referring to *unhealthy* foods, and advised restraint in general:

Also, avoid eating until you feel like popping—regular servings with fewer calories are more than enough. (DI29)

Listen to your body. Eat when you are hungry and stop when you are feeling full and satisfied. (DI11)

In these extracts, men’s eating *practices* are situated as unhealthy through an implied excess or lack of constraint. Healthy eating itself was depicted in ways which suggested it was difficult to eat healthily:

Each aisle of the grocery store contains hundreds of items that you have to scan through, and it’s not always clear which ones are the best for you. (DI28)

These days, it’s not always easy to follow a healthy diet. There are so many fast food and junk food options out there, a guy is tempted to just shrug, eat what he likes, and leave his health up to fate. (DI1)

Here, healthy eating was rendered difficult through the *effort* needed to either gain knowledge (DI28), or to resist easier temptations (DI1). Through such accounts, unhealthy eating was positioned as tempting for men, as well as the default way to eat (McDonald & Braun, 2022), and as something that required effort to resist. By engaging in a practice positioned as difficult and rendered more or less unnatural in the current context, and through avoiding the dual ease and temptation of unhealthy eating, men can demonstrate discipline and self-control. Within these representational frames, engaging in healthy eating can be understood to increase masculinity through demonstrating discipline and control—behaviors associated with hegemonic masculinity (Hinojosa, 2010; Mycek, 2018).

## Discussion and Conclusion

The contexts of neoliberal ideology and a claimed crisis in men’s health produce expectations that individual men will change “negative” health practices, and work to improve their health, through changes to practices like eating (e.g., Mahalik et al., 2007). As (healthy) eating is socially produced and traditionally in quite gendered ways, we wanted to understand the meanings and messages being made available to men around healthy eating, using online healthy eating advice as a widespread and important source of information and meaning-construction (Gough, 2006; Mackenzie & Murray, 2021). Given the prominence of online media in men’s health-related knowledge (Hodyl et al., 2020; Pew Research Center, 2003; Wang et al., 2021), this focus seemed important, and we attempted to produce a dataset of information a man searching for healthy eating advice would likely encounter. Our search terms for data generation were, however, based on considered and educated guesswork, in the absence of data on this. Although the dataset generated was rich and relevant, there is an opportunity for close ethnographic work that explores how men themselves actually engage with mediated health and healthy eating information.

Our research provided insight into what meanings and messages are being made available to men around healthy eating. We focused on the ways the data effectively sold healthy eating to men by drawing on hegemonic ideals of masculinity, constructing healthy eating as offering evidence of, and a route to, recognizably enhanced masculinity. Although a small-scale study, we demonstrated that ideas around masculinity and eating have persisted in media representations, despite sociocultural changes in media types and consumption practices, constructions and practices around health, and ideas of masculinity. Healthy eating was constructed as evoking an enhanced masculinity by providing the opportunity for men to use rational thinking and demonstrate self-control, or to gain increased masculinity through “enhanced” physical appearance, sexual performance, and youthfulness. Our analysis resonates with others who reported consistent reference to hegemonic ideals of masculinity when men justified their (conventionally unmasculine) eating practices, and when media represent men, health and healthy eating (e.g., Banyte et al., 2022; Gough, 2007; Mallyon et al., 2010; Mycek, 2018; Stibbe, 2004). This suggests that “healthy eating” remains a zone of potential trouble for men, something still at odds with normative masculinity, and that hegemonic idealized masculinity remains aspirationally powerful in Western societies (despite shifts in masculinity, e.g., Elliott, 2020).

One of the limitations of this study is that our analysis stops at the meanings made available to men. The consequent questions become how men themselves take up, use, or resist these meanings, and more broadly how men *themselves* construct their (un)healthy eating—with or without reference to media. There has been limited research exploring men’s constructions of healthy eating beyond a framework of barriers and motivators to healthy eating (see Ashton et al., 2015; Gough & Conner, 2006), but analyses around particular diet forms, or eating in certain contexts, suggest masculinity is a key concern for men in accounting for diet/ing (e.g., Mallyon et al., 2010; Mróz et al., 2011; Mycek, 2018). Further research on *how* exactly such media representations matter to men, and how men’s healthy eating is shaped by them, would offer insight beyond what our study could provide.

There are positive and negative (potential) implications of the constructions around healthy eating we identified. By resituating healthy eating as a masculinized practice, and constructing it as a practice that can enhance men’s masculinity, healthy eating may become more enticing to men who would otherwise eat less healthily. The use of masculinity tropes may therefore aid in reducing some of men’s negative health outcomes (Courtenay et al., 2002; Fagerli & Wandel, 1999; Gough, 2007; Wardle & Solomons, 1994). However, reiterating these constructions can reinforce the value of, and encourage alignment with, traditionally hegemonic ideals of (Western) masculinity, which are themselves problematic for health and well-being (e.g., Mahalik et al., 2007). As media are an important source of health information (e.g., Hodyl et al., 2020), it would be valuable to explore how successful mediated health promotion information that does not simply reinforce traditional masculinity tropes, and yet appeals “to men,” can be developed. Despite current media healthy eating information remaining apparently limited by traditional ideas, as masculinities “open up” (e.g., Elliott, 2020), especially for younger men, there is scope to utilize—and promote—different discourses and modes of masculinity in and through “healthy eating” advice—both in media and more official health promotion contexts.

What the focus on men and healthy eating in our dataset also retains is an emphasis on eating as *individual* practice and indeed personal “choice” (Dodds & Chamberlain, 2017)—which reproduces a neoliberalized account of (healthy) eating. Such media representation obscures the understanding of healthy eating as a socially embedded and facilitated practice, not (necessarily) within individual control, which materially constrains individual practice. We hope health promotion practitioners can draw on the insights of our analysis, and the questions we have raised, to develop information, and interventions, that both promote healthier eating for men, without *overly* reinforcing traditionally masculine tropes, and which also situate food and eating as relational, and contextual. Given the wider analysis of traditional modes of masculinity as health-limiting, such an orientation would also contribute to more general moves to promote men’s health.

## Notes

1. Neoliberalism in the form of social/economic policy also intersects with health, substantially, but this is not our focus here.

2. The intersections of masculinity with class, ethnicity, sexuality, and so on, mean the extent to which engaging with healthy eating practices might potentially be a threat to a man’s masculinity is variable and situated. There is no single or simple masculinity enacted through food (choices) (e.g., see Carroll et al., 2019).

3. The media have been used effectively to campaign against certain unhealthy practices and to promote healthy practices (Wakefield et al., 2010), but media “effects” are not always unequivocally positive (e.g., Escobar-Chaves et al., 2005).
